# Research progress of tumor‐derived extracellular vesicles in the treatment of malignant pleural effusion

**DOI:** 10.1002/cam4.5005

**Published:** 2022-07-21

**Authors:** Sijia Zhang, Leichong Chen, Yan Zong, Qianwen Li, Kuikui Zhu, Zhenyu Li, Rui Meng

**Affiliations:** ^1^ Cancer Center, Union Hospital, Tongji Medical College Huazhong University of Science and Technology Wuhan China

**Keywords:** immunotherapy, lung cancer, microparticle, malignant pleural effusion (MPE)

## Abstract

Vesicles, also known as “microparticles”, are vesicle‐like structures that are released outside the cell in a “sprouting” manner when the cytoskeleton is changed during cell activation or apoptosis, with a diameter of about 100–1000 nm, and are carriers of material information exchange between cells. Tumor‐derived extracellular vesicles can effectively deliver drugs to the nucleus of tumor stem cells, thus effectively killing them without toxic side effects. The underlying mechanism involves the soft nature of tumor stem cells that allows better uptake of vesicles, and the entry of drug‐carrying vesicles into lysosomes and facilitation of lysosomal movement toward the nucleus to deliver drugs to the nucleus. Drug‐loaded vesicles have unique advantages, such as low immunogenicity, homing targeting ability, and the ability to break through the physiological barrier to tumor therapy. Tumor‐derived drug‐delivery vesicles have entered clinical trials for the treatment of malignant pleural effusions. In this review, we summarized the progress of basic and clinical research on tumor cell‐derived drug‐loaded vesicles for the treatment of malignant pleural effusion in recent years.

## Graphical abstract

Extracellular vesicles, also known as "microparticles", are vesicle‐like structures that are released outside the cell in a "sprouting" manner when the cytoskeleton is changed during cell activation or apoptosis, with a diameter of about 100‐1000 nm, and are carriers of material information exchange between cells. Their physicochemical properties make them a promising nanomedicine for drug transport and release in cancer therapy, especially in the treatment of malignant pleural effusions (MPE). In this review, we summarized the progress of basic and clinical research on tumor cell‐derived drug‐loaded vesicles for the treatment of MPE in recent years and discuss the challenges and future directions.

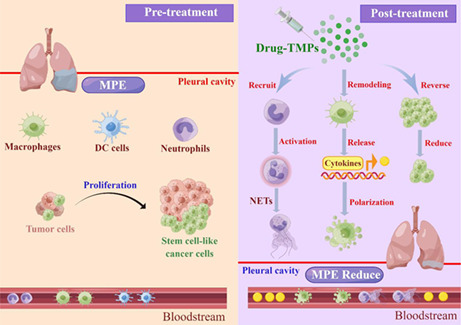

## 1 INTRODUCTION

Malignant pleural effusion (MPE), one of the most common complications of advanced lung cancer, is caused by pleural metastases with an incidence of up to 60%.^1^, ^2^ The median survival of patients with MPE is about 3–12 months.^2^ In recent years, with the in‐depth research on the pathogenesis of MPE, the clinical treatment means have been improved. At present, there are more clinical treatment methods for MPE, but the overall treatment efficiency is low. Among them, thoracic infusion of chemotherapeutic drugs for MPE is the most widely used method, but its efficacy is unsatisfactory and its long‐term toxic side effects are large.^3^ For example, bone marrow suppression, gastrointestinal reactions, fever, chest pain, and pleural fibrosis.^3^

Most of the drugs currently available for clinical treatment of MPE are non‐targeted, thus limiting the overall effect of the drugs. To solve this problem, drug delivery systems have emerged as a hot research topic in oncology therapy. An ideal drug delivery system can not only enhance drug efficacy, but also reduce the toxic side effects of drugs.^4^ It has been found that tumor cell‐derived drug‐loaded vesicles have the advantages of high cell affinity, high biocompatibility, high targeting, and specificity, and low immunogenicity, which can combine chemotherapy and immunotherapy and show a broad prospect in tumor therapy.^5^ In recent years, a lot of exploratory work has been carried out in the field of MPE treatment with drug‐loaded vesicles of tumor cell origin, which is reviewed in this review.

## 2 DEFINITION OF VESICLES

**FIGURE 1:**
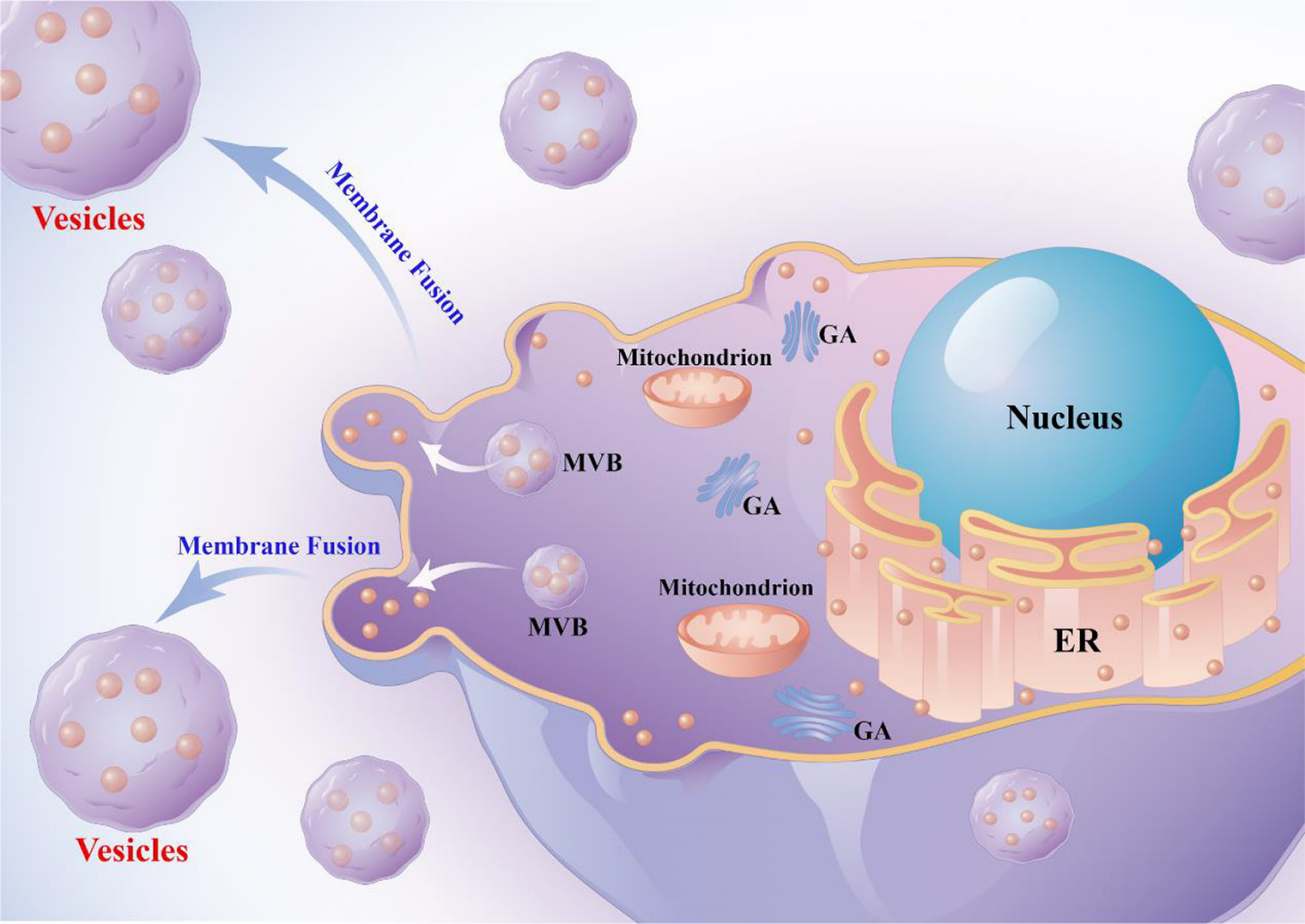
Extracellular vesicles are formed either by budding of the plasma membrane, in which case they are referred to as microvesicles, or as intraluminal vesicles (ILVs) within the lumen of multivesicular endosomes (MVEs). MVEs fuse with the plasma membrane to release ILVs that are then called vesicles. By Figdraw (www.figdraw.com) and Adobe illustrator.

Vesicles, also known as “microparticles”, are vesicle‐like structures with a diameter of about 100–1000 nm, which are the carriers of material information exchange between cells when the cytoskeleton is changed during cell activation or apoptosis and the cell contents wrapped by the cell membrane are released to the outside of the cell in a “sprouting” manner^6^ (Figure 1 **)**.

## 3 DEFINITION OF TUMOR CELL‐DERIVED DRUG‐LOADED VESICLES

**FIGURE 2:**
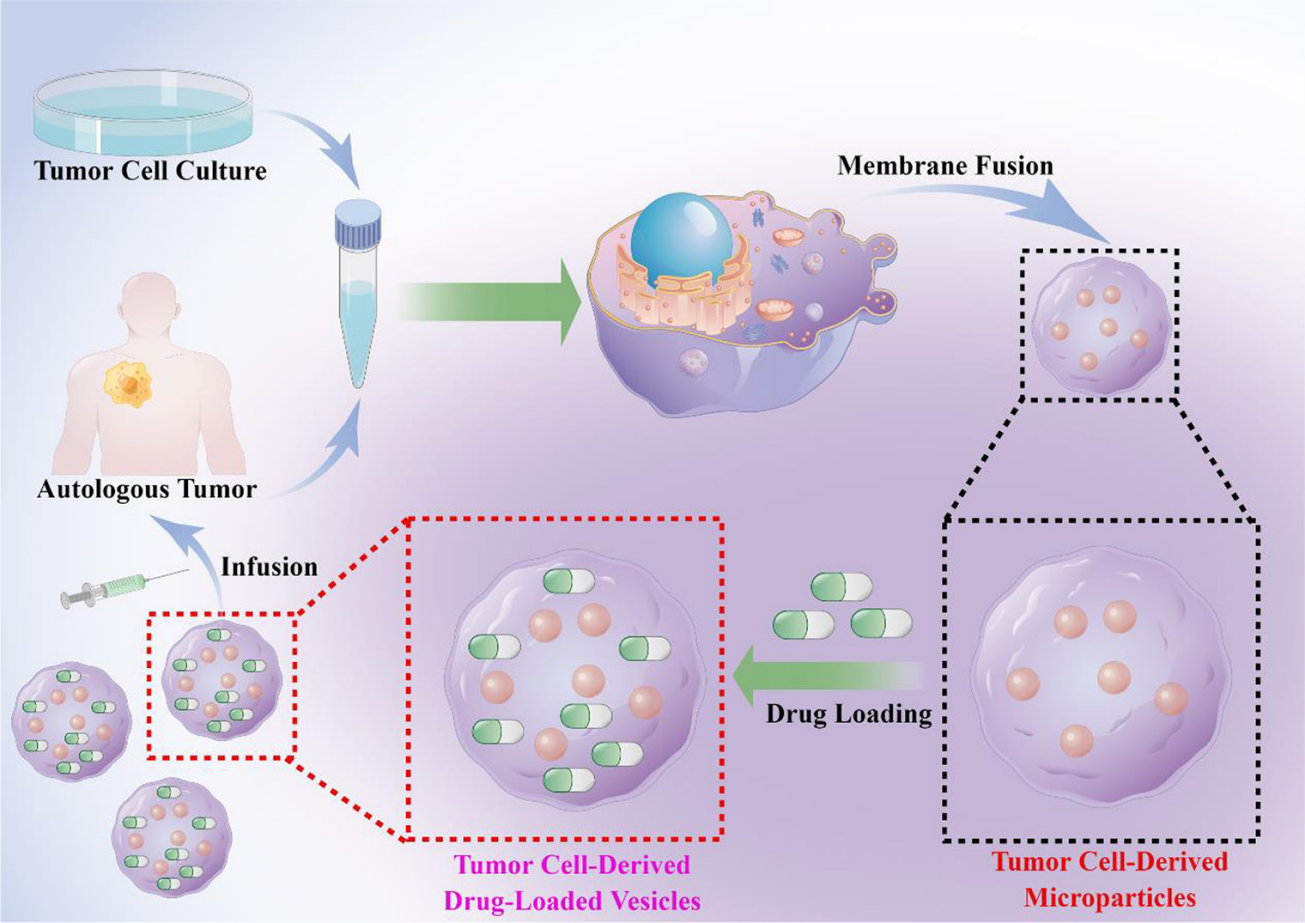
Tumor cell lines and autologous tumor cell‐derived vesicles have been specially processed to enable them to be combined with conventional chemotherapeutic drugs. Since the cell vesicles encapsulating conventional chemotherapeutic drugs are derived from tumor cells, they are easy to fuse with the cell membrane of tumor cells in the patient's body, and the anti‐tumor effect increases after infusion into the patient's body. By Figdraw (www.figdraw.com) and Adobe illustrator.

Tumor cell‐derived drug‐loaded vesicles (tumor cell‐derived microparticle, TMP) are specially processed to enable the vesicles to bind to conventional chemotherapeutic drugs.^7^ Since the cellular vesicles used to encapsulate conventional chemotherapeutic drugs are derived from tumor cells, they can easily fuse with the cellular membrane of tumor cells in patients, which is more conducive to the anti‐tumor therapeutic effect^8^ (Figure 2 **)**.

## 4 STRUCTURE OF VESICLES

Vesicles are formed by cells through a series of regulatory processes, such as “endocytosis‐fusion‐exocytosis”.^9^ They have a lipid bilayer, which prevents the contents of extracellular vesicles from being degraded by exogenous nucleases and proteases and facilitates the long‐distance transport of biomolecules.^10^ In addition, vesicles contain proteins, nucleic acids, lipids, coding, and non‐coding DNA and RNA, which are widely involved in biological processes such as intercellular information transfer, proliferation, migration, angiogenesis, and immune regulation, mediating tumorigenesis, development, and metastasis.^11^

## 5 VESICLES AND TUMORS

The search for tumor markers with high specificity and low toxicity has now become a hot topic in tumor diagnosis. Vesicles are expected to be a new tumor marker for early diagnosis of tumors.^12^ Because vesicles are widely found in eukaryotic cells, different types of tumor cells secrete different levels of vesicles, which helps to diagnose different types of tumors.^13^ In addition, the vesicles of different tumor cells carry specific protein and nucleic acid molecules, enabling the diagnosis of the corresponding diseases by detecting specific proteins and nucleic acids in vesicles.^13^ The bilayer membrane structure of vesicles can effectively protect the contents of proteins and nucleic acids from degradation by proteases in vivo, facilitating their detection as biomarkers.^12^ For example, miRNA is an evolutionarily relatively conserved non‐coding RNA molecule involved in post‐transcriptional regulation of gene expression. Vesicles containing miRNAs can avoid degradation due to the protective effect of lipid bilayers and are therefore often used as ideal molecular diagnostic markers.^14^ Brase et al. found that miRNA‐375 and miRNA‐141 were highly expressed in prostate patients and can be used as tumor markers for early screening of prostate cancer patients.^15^ In conclusion, with the advent of the era of precision medicine, the close relationship between vesicles and tumors deserves more in‐depth study to improve the diagnosis of tumors.

## 6 VESICLES CAN BE USED AS CARRIERS OF ANTITUMOR DRUGS

### 6.1 Vesicles carry a lot of proteins, genetic information, and biological substances

Although vesicles were initially thought to be cellular debris with no biological function, numerous studies have demonstrated the ability of vesicles to transmit biological information between cells.^16^, ^17^ It is currently believed that vesicles mediate intercellular signaling by two main mechanisms. First, vesicles act as signaling modules of the circulation, influencing cellular properties and target cell receptor activation by presenting membrane‐associated bioactive molecules.^18^ Second, vesicles can mediate signaling pathways that directly affect cellular activation, phenotypic modifications, and remodeling of cellular functions by delivering their contents.^19^ Including proteins, biologically active lipid molecules, or RNA, to the recipient cell.^19^ This transition is sufficiently enhanced during transient interactions or requires firm contacts, such as membrane assimilation or direct integration of vesicles into target cells. Vesicles express a large range of bioactive substances, membrane‐chimeric receptors, and adhesion molecules on their surface, providing the basis for specific interactions and information exchange with different target cells.^20^, ^21^ When vesicles are formed, the membranes of the vesicles are involved in some of the cytoplasmic components, and thus the vesicles also contain abundant cytokines, chemokines, enzymes, growth factors, and signaling proteins.^21^ Because of the large amounts of biological contents encapsulated in vesicles and their uptake by recipient cells after release from the parent cell, vesicles become the transmitters or carriers of cell‐to‐cell signal exchange and transmission. This feature of naturally existing message carriers or vectors in the organism provides unique conditions for the targeted transport of chemotherapeutic drugs.

During cancer development, tumor‐derived vesicles regulate dynamic and functional communication between cancer stem cells and cancer cells/the tumor microenvironment. Hu et al. have confirmed that vesicles derived from cancer‐associated fibroblasts have been shown to prime cancer stem cells and to contribute to drug resistance and chemoresistance through Wnt signaling pathway in colorectal cancer.^22^ Additionally, Richards et al. researched that the chemotherapeutic agent gemcitabine‐induced the upregulation and secretion of miR‐146a and Snail in cancer‐associated fibroblast‐derived vesicles, facilitating proliferation, and drug resistance in recipient pancreatic cancer cells.^23^ Hu et al. found that fibroblast‐derived vesicles that contain Wnt have been shown to contribute to chemotherapy resistance by restoring cancer stem cell characteristics in colorectal cancer cells in a Wnt/β‐catenin signal‐dependent manner, suggesting an important effect of tumor‐derived vesicles on drug resistance in cancer.^24^ These studies indicated the critical role of tumor‐derived vesicles in cell‐to‐cell communication during cancer development.

### 6.2 Vesicles are widely distributed in various body fluids and have good tolerance in vivo, which can maintain a long‐circulating half‐life and thus improve the efficacy

Tang K et al. encapsulated chemotherapeutic drugs by vesicles released from tumor cells, including methotrexate, cisplatin, and paclitaxel with vesicles released from mouse liver cancer ascites cells H22 and human‐derived ovarian cancer cells A2780, which showed good tumor‐killing effects in vivo and in vitro.^25^ Treatment of tumor cells with chemotherapeutic drugs, in which apoptosis was induced by UV light, resulted in the release of a large number of vesicles from the tumor cells, and the contents of the microparticles were examined for their corresponding drug concentrations by high‐performance liquid chromatography.^25^ It was found that H22 cells had a strong uptake of tumor cell microparticles, reaching 45% and 100% at 8 h and 24 h, while the uptake of tumor cell‐derived microparticles by CD3+ T cells was less than 5% at 8 h; the uptake of CD19+ B cells was also less than 20% at 8 h.^25^

### 6.3 Vesicles can transport substances across cell membranes to target cells, such as DC cell‐derived exosomes can transfer MHC‐like molecules to other DC cells and thus regulate tumor immunity

There is no doubt that DC cells play an antigen‐presenting role in T cell activation, and Zhang et al. showed that tumor cell‐derived vesicles can induce DC cell production of type I interferon through activation of the cGAS‐STING signaling pathway by tumor cell‐derived DNA molecules, thereby inducing DC cell activation and maturation.^26^ Tumor cell‐derived vesicles carry a large amounts of tumor antigens, however, tumor antigens can only be activated by DC cells through cross‐presentation pathways in the form of MHC‐ type I antigenic peptides presented to the cell membrane surface.^26^ Therefore, in today's increasingly individualized therapeutic approach, tumor cell‐derived vesicles can be used to prevent tumor recurrence, and their combination with DC cells is expected to be a new means of tumor immunotherapy.

### 6.4 Vesicles are capable of membrane modification to enhance cell‐specific targeting

Vesicles have different preferences for tumor cell subpopulations, with vesicles appearing to prefer undifferentiated tumor stem cells and less so for differentiated tumor cells.^27^ This seems to be related to the physical characteristics of tumor cells. Ma et al. study found that tumor stem cells are softer than differentiated tumor cells and their cell membranes are more easily deformed, thus facilitating their uptake of vesicles.^28^ Increasing the amount of cytoskeleton increased the stiffness of tumor stem cells, but their ability to take up microparticles decreased. In contrast, inhibiting the cytoskeleton and decreasing the stiffness of differentiated tumor cells enhanced the ability of differentiated tumor cells to take up microparticles.^28^ This selectivity of microparticles for tumor stem cells makes them more superior as drug carriers.

### 6.5 Unlike traditional synthetic liposomes, autologous vesicles are capable of loading drugs both in vivo and in vitro

Liposomes have received a lot of attention during the past 30 years as pharmaceutical.

carriers of great potential. The selective delivery of the anticancer agent doxorubicin in poly‐ethylene glycol (PEG) liposomes for the treatment of solid tumors in patients with breast‐carcinoma metastases, which have resulted in a subsequent improvement in survival.^29^, ^30^, ^31^ The same set of indications was targeted by a combination therapy comprising liposomal doxorubicin and paclitaxel or doxorubicin in PEG liposomes and carboplatin.^32^, ^33^ Clinical research showed the impressive effect of doxorubicin in PEG liposomes against unresectable hepatocellular carcinoma, cutaneous T‐cell lymphoma, and sarcoma.^34^, ^35^, ^36^ Liposomal lurtotecan was found to be effective in patients with topotecan‐resistant ovarian cancer.^37^ It would seem that liposomal drugs have a very promising future.

The 2013 Nobel Prize in Physiology and Medicine was awarded to three scientists from the United States and Germany, James E. Rothman, Randy W. Schekman, and Thomas C. Südhof, for their work on the intracellular vesicular transport system. Since then, the natural delivery vector “ microparticles” has been in the limelight. Zou et al. found that bacterial outer membrane vesicles (OMV) hybridized with tumor‐derived cell membranes (mT) to form new functional vesicles (mTOMV).^38^ Cellular experiments revealed that mTOMV enhanced the activation of innate immune cells and increased the specific lytic ability of T cells in homogeneous tumors.^38^ Mouse experiments showed that mTOMV efficiently accumulated in inguinal lymph nodes, which in turn inhibited lung metastasis.^38^ mTOMV has good biocompatibility and a simple preparation process, and has the ability to inhibit tumor growth and metastasis, offering great potential for clinical applications. In both the BALB/c mouse ascites cancer model and the nude mouse‐human ovarian cancer model, the drug‐laden vesicles showed significant cancer‐inhibiting effects and significantly prolonged the survival of the mice.^25^ And no significant adverse effects were produced during the vesicle treatment.^25^ In addition, clinical trials have been conducted on tumor cell‐derived vesicles loaded with methotrexate, and three types of vesicles are used in clinical administration, including oral, local, and intravenous administration. Oral administration is mainly used for patients with obstruction caused by upper gastrointestinal tumors, local infusion is for pleural effusion caused by lung cancer, and intravenous administration is for all types of solid tumors.

## 7 THE ACTION PRINCIPLES OF DRUG‐LOADED VESICLES

### 7.1 Reversing drug resistance of tumor stem cells and efficiently killing tumor stem cells

There is a group of tumor cells with high self‐renewal ability and prone to metastasis, called stem cell‐like cancer cells (SCLCC), which is highly drug‐resistant and is the main reason for tumor treatment failure.^39^ Liu et al. developed the tumor‐repopulating cell (TRC) technology, which is similar to SCLCC in that highly tumorigenic TRCs exist in hypoxic compartments far apart from solid tumor vessels, and the team treated patients with cisplatin‐resistant malignant pleural effusions with vesicles released from lung cancer cells wrapped in cisplatin, and tumor cells were significantly killed.^40^, ^41^, ^42^, ^43^ Moreover, in the mouse liver cancer malignant ascites model and lung cancer model, drug‐loaded vesicles can kill drug‐resistant TRC efficiently, which proves that drug‐loaded vesicles of tumor cell origin can reverse the drug resistance of TRC.^28^

### 7.2 Remodeling of tumor immune microenvironment

#### 7.2.1 Remodeling of macrophage polarization

Macrophages after activation included two main phenotypes: M1 type and M2 type.^44^ M2 type can mediate immunosuppression, angiogenesis, tumor metastasis, therapeutic resistance, and tumor stem cell formation.^45^ While M1‐type macrophages have opposite effects. Vacant Tumor microparticle induces macrophage conversion to M2 type via cGAS/STING/TBK1/STAT6 pathway, promoting M2 type macrophage proliferation and M1 type macrophage apoptosis, and M2 type macrophages, in turn, promote TRC proliferation, leading to tumor growth and metastasis.^46^ It has been shown that the combination of drug‐loaded vesicles and low‐dose radiotherapy can kill TRC, convert immunosuppressive M2‐type macrophages into M1‐type macrophages, reshape the immune microenvironment, and promote T cell‐mediated antitumor effects.^47^

#### 7.2.2 Recruit neutrophils

Tumor‐derived extracellular vesicles are taken up by tumor cells and induces their apoptosis. Apoptotic tumor cells can release chemokines CXCL1/CXCL2, recruit neutrophils in peripheral blood and induce them to change into N1‐type neutrophils with killing activity, thus completely removing tumor cells.^48^

#### 7.2.3 Activation of DC cells

Tumor‐derived extracellular vesicles can carry tumor self‐antigens and has strong immunogenicity.^49^ Using DC cells as a carrier, tumor‐derived extracellular vesicles can be efficiently taken up by DC cells.^49^ The DNA fragment carried by tumor‐derived extracellular vesicles activates the cGAS‐STING signaling pathway to induce DC cells to produce IFN‐I, which in turn promotes the activation and maturation of DC cells, enhances their antigen presentation, and promotes anti‐tumor immune response.^26^, ^50^

#### 7.2.4 Stimulate T lymphocytes

**FIGURE 3:**
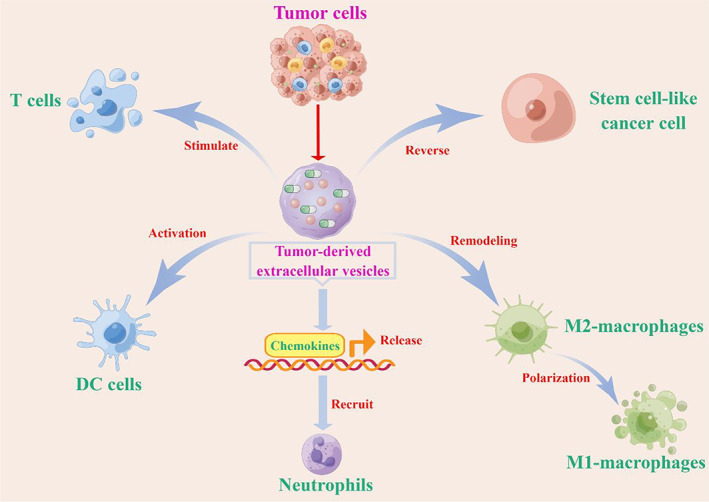
The action principle of drug‐loaded vesicles. Tumor cell‐derived exosomes may promote immune cells, as follows. (1) Reversing drug resistance of tumor stem cells and efficiently killing tumor stem cells. (2) Remodeling of macrophage polarization. (3) Release chemokines CXCL1 and CXCL2 and recruit neutrophils. (4) Activation of DC cells. (5) Stimulate T lymphocytes. By Figdraw (www.figdraw.com) and Adobe illustrator.

Tumor‐derived extracellular vesicles can stimulate CD4+ T cells to release IL‐2, stimulate CD8+ T cells to release IFN‐γ, and finally activate helper T lymphocytes and cytotoxic T lymphocytes, increase the percentage of immune effector cells, induce anti‐tumor immunity, and reverse the tumor immunosuppressive microenvironment^51^, ^52^ **(**Figure 3 **)**.

### 7.3 Blocking drug efflux from tumor cells

Tumor cells excrete chemotherapeutic drugs extracellularly is one of the reasons for tumor drug resistance.^28^ The uptake of drug‐carrying vesicles by tumor cells inhibits the efflux of chemotherapeutic drugs from tumor cells by down‐regulating the expression of multidrug resistance protein P‐glycoprotein in ABC transporter protein, which maintains the intracellular action of drugs and increases the sensitivity of chemotherapeutic drugs.^25^, ^26^, ^27^, ^28^

## 8 UPTAKE MECHANISM OF DRUG‐CARRYING VESICLES

### 8.1 Classical pathway

In vivo cells take up extracellular substances through endocytosis. During endocytosis, the cell membrane is deformed and bent inward to wrap extracellular substances into early endosomes, which are then transported to lysosomes via late endosomes. Huang Bo et al. researched the co‐cultured tumor‐derived extracellular vesicles with DC cells and macrophages respectively, and labeled the early endosomes, late endosomes, and lysosomes of the cells.^28^ Under confocal microscopy, tumor‐derived extracellular vesicles were observed to co‐localize with early endosomes, late endosomes, and lysosomes of macrophages and DC cells, indicating that macrophages and DC cells took up drug‐laden vesicles through classical endocytosis.

### 8.2 Non‐classical pathway

Tang et al. researched the co‐incubated tumor‐derived extracellular vesicles with tumor cells and labeled lysosomes, endoplasmic reticulum, Golgi apparatus, and endosomes.^25^ Under confocal microscopy, tumor‐derived extracellular vesicles were observed to co‐localize only with lysosomes, which confirmed that different types of cells have different uptake patterns. Tumor cells take up drug‐loaded vesicles through a non‐classical pathway.^28^ Drug‐loaded vesicles fuse with lysosomes to form drug‐loaded lysosomes, which increases the pH in the lysosomes and causes the drug‐loaded lysosomes to move along microtubules and accumulate around the nucleus, releasing the drug in the nucleus and maximizing sensitization to drug efficacy and thus killing tumor cells.^28^

Molecular recognition and uptake at bilayer vesicles provide important insight into recognition processes at biological membranes, which are generally mediated by carbohydrate‐protein interactions. It is increasingly evident that the density and distribution of receptors and ligands in the membrane, their clustering by cofactors, and the presence of competitors and inhibitors are critical to the regulation of biological recognition at membranes. Furthermore, Clayton A et al. have confirmed that most vesicles carrying PD‐L1 can bind to PD‐1 expressed on activated T cells to suppress T cell receptor (TCR)‐related pathways and trigger immunosuppressive effects on T cells.^53^ Fas (CD95), also known as death receptor, is a transmembrane receptor existing on the cell membrane.^54^ When vesicles carrying FasL bind to Fas on the T cell surface, the transmembrane transmission of apoptosis signals is initiated and subsequently induces T cell apoptosis.^54^ Here, the two pathways depend on ligand recognition between vesicles and recipient cells.

## 9 BASIC RESEARCH ON MPE TREATMENT BY TUMOR‐DERIVED EXTRACELLULAR VESICLES

### 9.1 Macrophages are related to the formation of MPE

In the tumor microenvironment, M2‐type macrophages lead to tumor immune escape by promoting tumor angiogenesis. Studies have shown that the degree of infiltration of M2‐type macrophages is positively correlated with the ability of tumor cells to invade and migrate.^55^ Therefore, by removing M2 macrophages, drug‐loaded vesicles can efficiently treat MPE. Methotrexate‐encapsulated tumor cell‐derived vesicles were found to control the progression of MPE by targeting tumor cells. It not only directly kills tumor cells, but also can be phagocytosed by macrophages to promote their apoptosis in the treatment of MPE.^56^ When macrophages are removed, methotrexate‐encapsulated tumor cell‐derived vesicles can control the progression of MPE more effectively.^56^

### 9.2 Irradiated tumor cell‐derived microparticles mediate tumor eradication via cell killing and immune reprogramming

The irradiated tumor cell‐released microparticles (RT‐MPs), which induce broad antitumor effects and cause immunogenic death mainly through ferroptosis. Wan et al. used a mouse model of MPE, demonstrated that RT‐MPs polarized microenvironmental M2 tumor‐associated macrophages (M2‐TAMs) to M1‐TAMs and modulated antitumor interactions between TAMs and tumor cells.^57^ Following internalization of RT‐MPs, TAMs displayed increased programmed cell death ligand 1 (PD‐L1) expression, enhancing follow‐up combined anti‐PD‐1 therapy that confers an ablative effect against MPE and cisplatin‐resistant MPE mouse models. Immunological memory effects were induced.^57^

## 10 CLINICAL STUDIES OF TUMOR‐DERIVED EXTRACELLULAR VESICLES FOR MPE

### 10.1 Methotrexate‐encapsulated tumor cell‐derived vesicles for MPE

MPE is one of the most common complications of lung cancer after progression. Thoracic infusion of methotrexate‐encapsulated tumor cell‐derived vesicles can significantly reduce the recurrence of MPE and relieve patients' symptoms.^58^ Guo et al. reported that the efficiency of thoracic infusion of methotrexate‐encapsulated tumor cell‐derived vesicles for MPE reached 90.91%, patients' symptoms and quality of life improved significantly, and no significant toxic side effects were observed.^56^

### 10.2 Cisplatin‐coated tumor‐derived extracellular vesicles (tumor‐derived extracellular vesicles derived from A549 cells, Cis‐tumor‐derived extracellular vesicles) for MPE

Ma et al. found that thoracic infusion of Cis‐tumor‐derived extracellular vesicles not only killed tumor cells in pleural effusion, but also significantly improved patients' quality of life.^28^

## 11 THE DIFFICULTIES THAT TUMOR‐DERIVED DRUG‐LOADED VESICLES MIGHT ENCOUNTER IN CLINICAL APPLICATION

### 11.1 Lack of accurate and effective vesicle separation methods

So far, five types of exosome separation strategies have been reported, including ultra‐speed centrifugation, ultrafiltration, immunoaffinity capture, and microfluidic techniques, with unique sets of advantages and disadvantages for each technique (Table 1).

**TABLE 1:** Methods currently used to separate vesicles

Separation methods	Principles	Advantages	Disadvantages
Sequential ultracentrifugation	Centrifugal force changes the settling velocity of particles with different density and size	Low cost; Ideal for large scale preparations;	High equipment requirements; Time consuming; Protein aggregation; Low portability;
Gradient ultracentrifugation	Separation based on density differences between particles and media	Having a high purity; Separating subpopulations of vesicles;	High equipment requirements; Time consuming; Labor intensive; Not suitable for small volume diagnosis; Low portability;
Ultrafiltration	Filter membranes that have a defined size‐exclusion limit or molecular weight threshold	Fast procedure; Low equipment cost; Good portability;	Moderate purity; Potential deterioration induced by shear stress; Possible loss due to clogging and membrane trapping;
Immunoaffinity capture	Based on specific binding between exosome markers and immobilized antibodies	Separates vesicles of a specific source; High purity vesicles; Simple to use;	High‐cost antibodies; Low processing volume and yields; Extra step for exosome elution may damage native exosome structure;
Microfluidics‐based techniques	A combination of different factors including size, density, and immunoaffinity	Portable, highly efficient, and cost‐effective; Easily automated and integrated into diagnosis	Low sample capacity;

Ultracentrifugation has become the most accepted and used standard protocol for vesicle isolation and purification with its dual‐dimensional combination of differential sedimentation based on size separation and isodensity gradient centrifugation based on density differentiation. The prevailing method for confirming vesicle purity relies on the ratio of particle count to protein content as a criterion for assessing vesicle purity. However, this method can neither detect vesicles smaller than 70 nm nor distinguish lipoprotein or protein aggregates that are similar in size to vesicles, resulting in inaccurate isolation of vesicles.^59^ Therefore, exploring optimal isolation and purification method for subsequent analysis, detection and clinical application is crucial for the widespread clinical use of drug‐laden vesicles.

### 11.2 The identification method of vesicles has a long way to go

Current vesicle detection methods mainly include scanning electron microscopy, atomic force microscopy, dynamic light scattering techniques, nanoparticle tracking analysis (NTA), flow cytometry, and ELISA. ELISA and flow cytometry are the more commonly used methods due to their high throughput, short time, and simple operation. However, each of these assays has its own limitations and needs to be continuously optimized in clinical applications. Whole transcriptome analysis of vesicles is currently showing great promise. Therefore, for the clinical application of RNA analysis of vesicles, it is important to develop vesicle‐specific protocols and achieve deeper coverage than that typically used for sequencing tumor tissue.

In addition, a light microscopic single extracellular vesicle analysis (SEA) technique under a light microscope that allows robust, multi‐component protein biomarker measurement in a single vesicle. In this method, extracellular vesicles are immobilized inside a microfluidic chamber, immuno‐stained, and imaged. The SEA technology may be a powerful tool to study various extracellular vesicle types in the future, providing rich data sets about the expression heterogeneity of biomarkers, marker make‐ups, and the presence of extracellular vesicle subpopulations.

In conclusion, it will be a hot research topic for drug‐loaded vesicles in the field of oncology therapy to take advantage of their high affinity, biocompatibility, targeting and specificity, and low immunogenicity, while attenuating or avoiding their side effects.

## 12 MECHANISM OF DRUG‐LADEN VESICLES FOR MPE

Xu et al. found that methotrexate‐encapsulated tumor cell‐derived vesicles were able to recruit neutrophils to MPE in lung cancer patients, and the neutrophils exhibited an anti‐tumor phenotype, not only killing tumor cells but also releasing neutrophil extracellular trap networks (NETs) to seal damaged endothelial cells, thus effectively treating MPE.^48^ It was found that the release of chemokines CXCL1 and CXCL2 were found to play an important role in the attraction of neutrophils to MPE.^48^ In addition, drug‐coated tumor‐derived extracellular vesicles were able to convert M2‐type macrophages toward M1‐type macrophages. In addition, methotrexate‐encapsulated tumor cell‐derived vesicles are able to indirectly affect neutrophil activation via the macrophage pathway, where macrophages release soluble factors that induce neutrophil polarization to type N1 upon uptake of Methotrexate‐encapsulated tumor cell‐derived vesicles. Methotrexate‐encapsulated tumor cell‐derived vesicles recruited neutrophils release NETs in MPE.^47^, ^48^ NETs are fibrillar networks composed of chromatin and serine proteases that play important role in tumors. NETs have been found to promote tumor growth and metastasis.^48^ However, this study found that NETs have an inhibitory effect on tumors and that NETs can activate DNA fibers, histone structural proteins and proteasomes to exert anti‐tumor effects. In the MPE of lung cancer patients, NETs were found to surround tumor cells, inhibit tumor cell migration and promote tumor cell killing by neutrophils.^48^ In addition, NETs can interfere with vascular leakage, thus effectively treating MPE.

**FIGURE 4:**
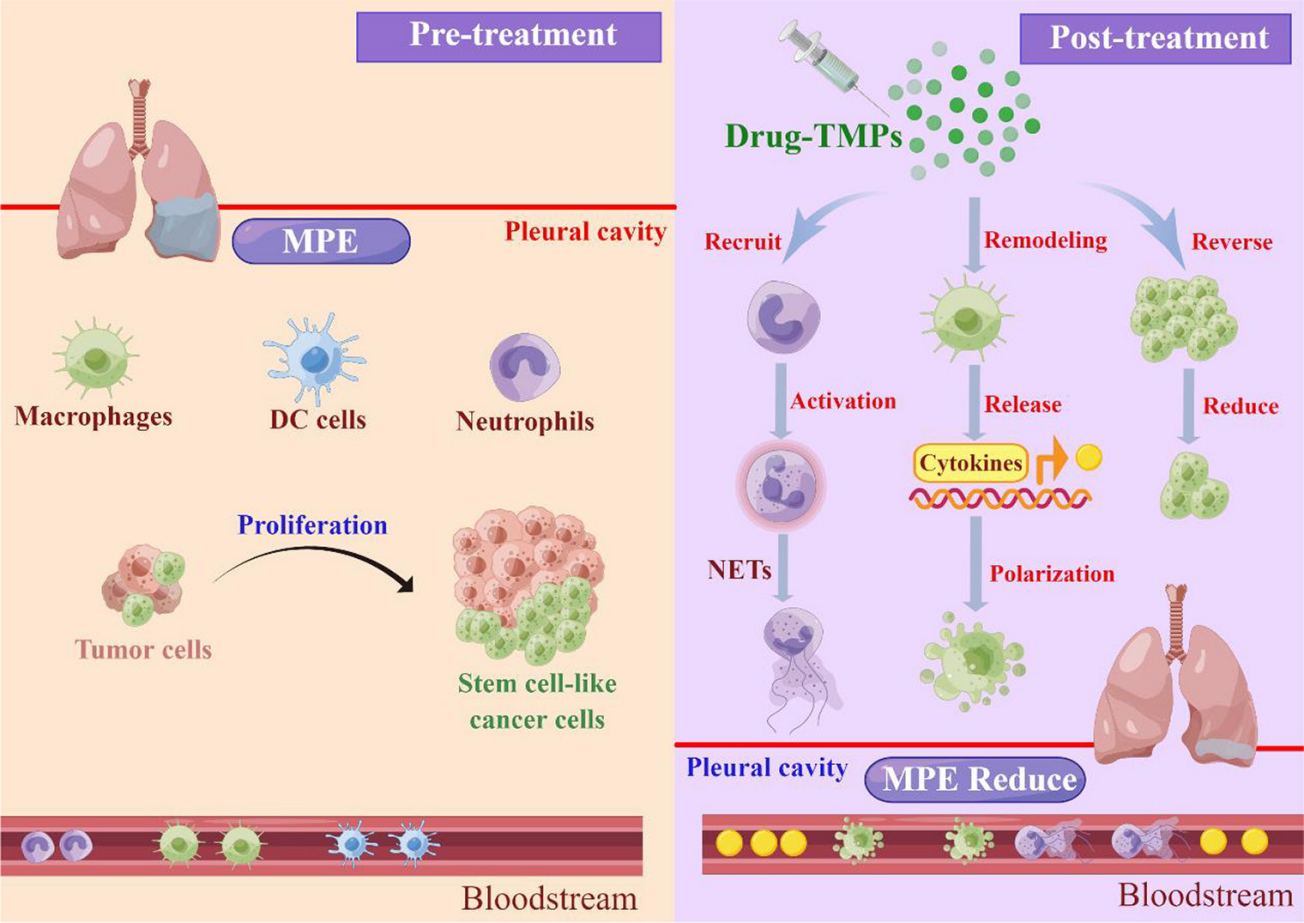
Schematic diagram for TMPs treatment of MPE. Following the injection into the MPE via pleural catheter, TMPs are taken up by and kill tumor cells. TMPs may reverse drug resistance of tumor stem cells and efficiently killing tumor stem cells. TMPs are taken up by and induce macrophages to release cytokines, thus recruiting neutrophils to the MPE. Neutrophils are activated in TMPs‐remodeled malignant environments. Activated neutrophils launch the second wave of killing tumor cells. Neutrophils release NETs to seal off the damaged endothelium, facilitating the regression of the MPE. By Figdraw (www.figdraw.com) and Adobe illustrator.

In conclusion, drug‐laden vesicles serve as a unique approach to enhance the recruitment of neutrophils and the polarization of M1‐type macrophages leading to enhanced antitumor effects and thus effective treatment of MPE^48^ (Figure 4 **)**.

## 13 DRUG‐LADEN VESICLES COMBINED WITH IMMUNE CHECKPOINT INHIBITORS FOR MPE

Not only do drug‐laden vesicles lead to regression of MPE, but notably, the primary tumor is controlled in some lung cancer patients. Suggesting that the body's antitumor immune response may be activated by drug‐loaded vesicles. Therefore, the combination of drug‐loaded vesicles with current tumor immunotherapies, such as PD‐1/PD‐L1 inhibitors or CAR‐T therapies, is expected to achieve synergistic treatment of both primary and distant tumors.

## 14 CONCLUSIONS AND PERSPECTIVES

Most patients with thoracic malignancies are complicated by MPE, especially lung cancer, breast cancer and malignant lymphoma. Once combined with MPE, the median survival time is only 3–12 months.^59^ Currently, the conventional regimen for MPE is mainly based on diuresis, salt restriction, puncture and aspiration, intraluminal drainage, chest infusion of chemotherapeutic drugs, and surgery. However, rapid relapse and high toxic side effects lead to limited efficacy of the above treatments. Therefore, the exploration of drugs and treatment strategies with low immunogenicity, homing targeting ability, breaking the physiological barrier of tumor treatment, and high safety is the key to obtain survival benefits for MPE patients, which has important scientific significance and clinical value. Tumor immunotherapy is currently a hot topic in tumor treatment. Drug‐carrying vesicles combined with immunotherapy can effectively deliver drugs to target cells, reverse the drug resistance of tumor stem cells, reshape the immunosuppressive microenvironment of tumor, and increase the efficacy of immunotherapy, showing great potential. So that vesicles are important mediators of the immune system, and their immunotherapeutic or combination therapy potential could be exploited in many ways. Tumor‐derived extracellular vesicles have been shown to regulate immune responses in various ways, but this may change when it is injected, depending on route, dose and modifications. It contains antigens that could be presented, either directly on the tumor cell surface MHC class I, or through appropriate pathways via the recipient APCs. However, by altering, for example, cell targeting properties, remove the membrane protein components or cargo, tumor‐derived extracellular vesicles might be considered for a promising therapy and even used in engineering production. In addition, how to enhance the targeting of tumor‐derived vesicles and overcome the treatment resistance caused by related information has important clinical value. We provide the following ideas for the design and pre‐clearance methods of drug‐loaded vesicles. First, the exocrine surface is modified with targeted molecules, such as targeted peptides, antibodies, and so on. Second, the tumor‐derived vesicles loaded with magnetic nanoparticles were prepared to have the ability of magnetic targeting. Third, through the transformation of mother cells, the expression of exocrine targeting ligands was increased and its targeting ability was improved. Last, the active targeting ability of tumor‐derived vesicles was improved by fusion of exosomes with liposomes carrying targeted molecules. There are no prospective Phase III clinical studies to confirm the efficacy and safety of tumor‐derived extracellular vesicles in treating MPE, but retrospective studies and Phase I pre‐tentative clinical studies have confirmed that tumor‐derived extracellular vesicles can improve the disease control rate of MPE with good safety and can significantly improve the prognosis of patients.

In conclusion, tumor‐derived extracellular vesicles have high objective efficacy, low adverse effects, good patient tolerance and can significantly improve patients' quality of life in the treatment of MPE. However, the advantages and disadvantages of autologous tumor cells and tumor cell lines as the parent cells for tumor‐derived extracellular vesicles preparation need to be further clarified, and the occurrence of immune rejection of allogeneic tumor‐derived extracellular vesicles should be sought. In addition, it would be valuable to further explore biomarkers to screen for populations of preference for MPE patients to benefit from tumor‐derived extracellular vesicles therapy. Can the combination of tumor‐derived extracellular vesicles and immune checkpoint inhibitors improve patient outcomes in oncology? Although such studies are still in the exploratory stage, tumor‐derived extracellular vesicles have shown promising applications in tumor immunotherapy.

## AUTHOR’S CONTRIBUTION

Rui Meng conceived the study. Sijia Zhang participated in writing. Leichong Chen collected the materials for this study. Yan Zong and Qianwen Li prepared the figures. Kuikui Zhu and Zhenyu Li revised the manuscript. All authors approved the final manuscript.

## FUNDING INFORMATION

The authors would like to thank supports of the Foundation for Fostering Key Talents from Middle‐aged and Young Medical Personnel in Wuhan (2016), CSCO Cancer Research Fund (NO. Y‐2019Genecast‐061 and NO. Y‐sy2018‐018), 2019 Wu Jieping Medical Fundation‐ Xinda Cancer Research Fund to Rui Meng.

## CONFLICTS OF INTEREST

The authors declare no commercial or financial conflict of interest.

## Data Availability

N/A
